# Adaptative Variation in a Neotropical Dung Beetle: Females and Gamma Males Present Tunneler Morphology, While Beta and Alpha Males Present Wing Morphology for Velocity

**DOI:** 10.1002/ece3.70457

**Published:** 2024-11-24

**Authors:** Pedro Henrique de Oliveira Ribeiro, Nicholas Ferreira Camargo, Marina Regina Frizzas

**Affiliations:** ^1^ Graduate Program in Ecology University of Brasília Brasília District Federal Brazil; ^2^ Department of Zoology University of Brasília Brasília District Federal Brazil; ^3^ Department of Ecology University of Brasília Brasília District Federal Brazil

**Keywords:** flight biomechanics, geometric morphometrics, phenotypic integration, polyphenism, sexual dimorphism

## Abstract

In evolutionary terms, plastic traits can covary, providing adaptive value by compensating for certain characteristic costs or enhancing fitness. This combination of traits is expected in species with significant intrapopulation ecological variation, like insects. In the Phanaeini tribe, males and females have distinct nesting behaviors, and the alpha, beta, and gamma morphotypes of trimorphic males display different reproductive strategies. Although phenotypic integration of wings and protibiae associated with horn size occurs in dung beetles, this study focuses on the morphological and functional variation of these and other structures due to behavioral differences and reproductive strategies between sexes and male morphotypes. We used a geometric and functional morphometric approach to investigate the variation in plasticity of structures (clypeus, protibia, elytra, and membranous wing), their integration with horn size in males, and flight biomechanics in the trimorphic dung beetle *Oxysternon palemo* (Scarabaeinae: Phanaeini). Comparing different sexes and morphotypes, we tested for significant differences associated with various reproductive and behavioral strategies. Adaptive morphological differences were found in all structures in at least three of the four groups (females and alpha, beta, and gamma males), along with clear sexual dimorphism in the protibia. In alpha males, fossorial structures enhance resource partitioning and confrontations, whereas in females and gamma males, these structures optimize digging and tunnel mobility. We also found integration between the size of pronotal horns and clypeus shape, and between head horn size and wing shape. The variation in elytra morphology, covariation between membranous wings and horns, and flight biomechanics results suggests different reproductive investment and foraging strategies among groups. Alpha and beta males invest in intense flights and rapid resource colonization, whereas gamma males exhibit slower, low‐energy flights with greater reproductive investment. We discuss how trade‐offs between dispersal and reproduction in polyphenic insects shape adaptive variation through plasticity in dung beetle morphotypes.

## Graphical abstract

In evolutionary terms, plastic traits can covary, providing adaptive value by compensating for certain characteristic costs or enhancing fitness. We used a geometric and functional morphometric approach to investigate the variation in plasticity of structures (clypeus, protibia, elytra, and membranous wing), their integration with horn size in males, and flight biomechanics in the trimorphic dung beetle. The variation in elytra morphology, covariation between membranous wings and horns, and flight biomechanics results suggest different reproductive investment and foraging strategies among groups.

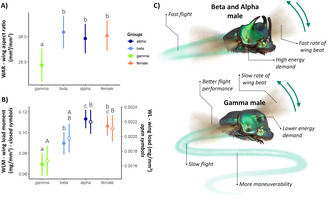

## 1 Introduction

Phenotypic plasticity is the ability of a single genotype to alter the physiology, morphology, and behavior of an organism in response to environmental stimuli (Pigliucci 2005). It can be either adaptive or non‐adaptive, depending on whether the phenotypes resulting from the environment are closer to or farther from the local optimum (Schmalhausen 1949). However, in organisms with appropriate genetic variability, phenotypic plasticity is expected to evolve in populations facing predictable environmental changes (West‐Eberhard 1989). From a holistic perspective, plastic traits can covary and interact through phenotypic integration (Pigliucci 2003). This integration can evolve and acquire adaptive value when there are compensations associated with the costs of certain traits or improvements in their fitness, thereby optimizing or correcting traits. Therefore, natural selection is expected to favor the co‐occurrence or combination of functional and morphological traits through correlational selection, especially in species with extensive ecological variation [e.g., nematodes (Ragsdale et al. 2013), cichlids (Kocher et al. 1993), anole lizards (Losos et al. 1998), and frogs (Ledón‐Rettig and Pfennig 2011)].

Given the high intrapopulational ecological variation in insects, these organisms often exhibit phenotypic integrations associated with polyphenisms (Simpson, Sword, and Lo 2011). Among the types of phenotypic plasticity, polyphenism is the expression of different and discrete phenotypes generated from a single genotype. Key examples of this phenomenon in insects include distinct developmental stages in holometabolous insects, seasonal phenotypes in aphids and lepidopterans, alternative dispersal strategies in aphids and locusts, castes in eusocial hymenopterans, and alternative reproductive strategies in dung beetles (Simpson, Sword, and Lo 2011). In the last example, two threshold mechanisms of body growth, resulting from immature nutrition, interact to produce three male morphotypes (alpha, beta, and gamma) with distinct reproductive tactics. Alpha males are territorial, aggressive, and defend tunnel entrances with long horns; beta males are subdominant and compete less efficiently with alpha males, having smaller bodies and shorter horns; gamma males lack horns and gain stealthy access to monitored burrows through adjacent tunnels (Emlen 1997; Moczek and Emlen 2000; Rasmussen 1994). In this reproductive system, smaller males often compensate for their lower fighting ability by investing in sperm competition (e.g., larger testis mass, ejaculation volume, and sperm size) (Simmons, Tomkins, and Hunt 1999; Simmons, Emlen, and Tomkins 2007), whereas larger males frequently invest in dispersal (e.g., higher flight speed and wing allometry) (Hongo 2010; Rohner, Macagno, and Moczek 2020).

Besides different investment patterns, phenotypic integration between horn production and functionally important structures such as wings and legs occurs in polyphenic dung beetle males (Rohner, Macagno, and Moczek 2020). Similarly, in Odonata, males of species with alternative territorial tactics exhibit morphological differences in wings (Kaunisto and Suhonen 2023; Outomuro et al. 2014). However, other structures may also integrate with these phenotypes, and little is known about how morphological variation reflects the functionality of these traits and consequently their reproductive success. Given the high competition among dung beetles, their reproductive success depends on the rapid location of resources and mating with conspecifics (Scholtz, Davis, and Kryger 2009). While fossorial structures (e.g., protibiae and clypeus) facilitate rapid burrow excavation and resource relocation, membranous wings enable foraging and dispersal in search of resources and/or conspecifics. Additionally, structures such as elytra reflect the shape of the abdomen, providing physical and thermal protection (Goczał and Beutel 2023), but are less studied. The functional perspective provided by flight biomechanics factors also allows a good estimation of flight patterns, dispersal, and foraging in beetles, especially in dung beetles (deCastro‐Arrazola et al. 2023; Ospina‐Garcés et al. 2018; Tocco, Dacke, and Byrne 2019). Wing proportion indicates flight maneuverability and speed (Dudley 2002), and wing loading moment indicates wing beat rate and flight energy expenditure (Ospina‐Garcés et al. 2018). Thus, structures may vary both morphologically and functionally, depending on the behavioral differences and reproductive strategies of the species.

In the present study, we used a geometric and functional morphometric approach (i.e., flight biomechanics indices) to investigate the variation in plasticity and biomechanics of ecologically important structures for dung beetles (i.e., clypeus, protibia, membranous wings, and elytra) in a trimorphic male dung beetle species, *Oxysternon palemo* (Scarabaeinae: Phanaeini). Additionally, we tested possible phenotypic integrations (covariation) between the relative horn size and the shape of these structures in the species. In the Neotropical region, species of the tribe Phanaeini are the main competitors (Price and May 2009) and exhibit the highest average dispersal rate (da Silva and Hernández 2015). In these species, as well as in other horned dung beetle species (e.g., Onthophagini), males and females cooperate in nest building after pairing (Hunt and Simmons 2002; Palestrini and Rolando 2001; Simmons and Ridsdill‐Smith 2011). While females excavate the tunnels, males partition and supply these nests with resources, providing competitive advantages over other individuals or species (Price and May 2009). Due to this behavior, in addition to the possible differences between male morphotypes, there may also be morphological and functional variations associated with behavioral differences between males and females. However, these variations have not yet been tested.

We hypothesize that due to the different selective pressures arising from behavioral differences and differential investment between males and females, as well as among male morphotypes, there will be adaptive morphological differences between individuals from natural populations of *O. palemo*. We predict that fossorial structures (i.e., clypeus and protibia) of smaller males and females will be morphologically more similar due to selective pressures associated with digging and tunnel mobility. Similarly, we expect that dispersal and defense structures (i.e., wings and elytra), along with flight biomechanics indices, will primarily reflect the reproductive and dispersal investment patterns among morphotypes. We anticipate that larger males will be better adapted for intense flights with higher energy costs, whereas smaller males will exhibit economical and low‐intensity flights. Furthermore, we expect phenotypic integration between the horns and the shape of the structures, particularly as compensations associated with the costs of armament production.

## 2 Methodology

### 2.1 Study Species

**FIGURE 1:**
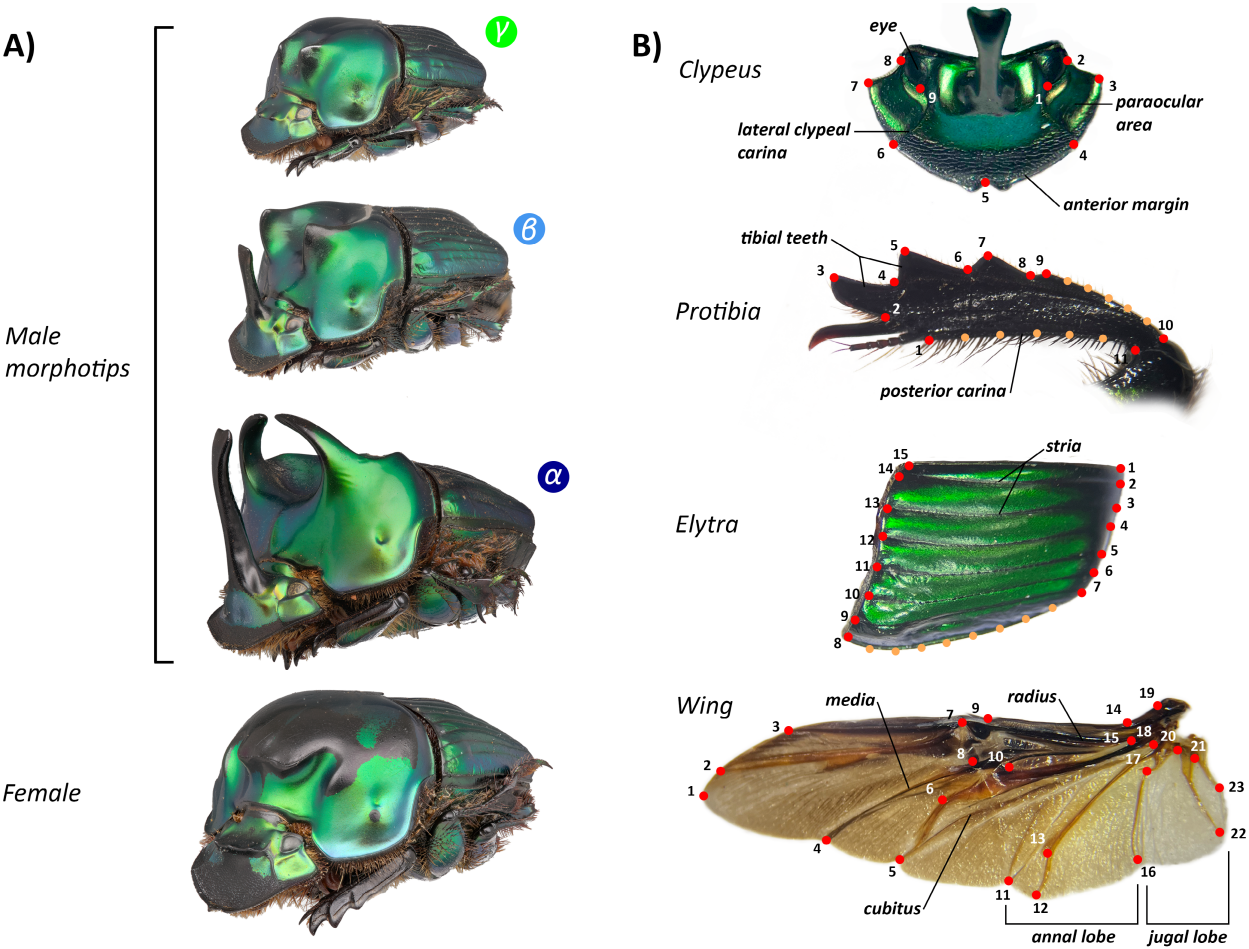
(A) Morphotypes alpha, beta, and gamma of trimorphic males and females of the dung beetle *Oxysternon palemo* (Scarabaeinae: Phanaeini); (B) Two‐dimensional morphometric measurements performed for all individuals (*n* = 155): Anatomical landmarks (red circles) and semi‐landmarks (orange circles) on the clypeus (9 landmarks), protibia (10 anatomical landmarks), elytra (15 landmarks and 8 semi‐landmarks), and wing (23 landmarks). The distance between points 1 and 23 was used to measure the length of the wing.

To assess the morphometric variation between sexes and the three male morphotypes, we used the model species *O. palemo* due to its high abundance in Brazilian savannas and good representation in collections. This species measures 12 to 18 mm in length and 8 to 12 mm in width and exhibits a greenish coloration. It is typical of Neotropical savannas, especially in the Cerrado *sensu stricto* of central Brazil, the eastern grasslands of Bolivia, and northern Paraguay at estimated altitudes of 200 to 1200 m (Maldaner, Costa‐Silva, and Vaz‐de‐Mello 2024; Ribeiro, Togni, and Frizzas 2022). This species is a typical tunneler, generalist, primarily coprophagous, and diurnal, being most abundantly collected from September to June (Edmonds and Zídek 2004; Ribeiro, Togni, and Frizzas 2022). *Oxysternon palemo* exhibits clear sexual dimorphism, with medium (beta) and large (alpha) males having head horns, whereas small (gamma) males and females lack head horns. The species also has pronotal horns in all male morphotypes, but not in females (Figure 1A).

### 2.2 Data Collection

To evaluate the morphometric variation between sexes and male morphotypes, we randomly selected 115 males and 40 females of *O. palemo* from the dry collection of the Entomological Collection of the Department of Zoology at the University of Brasília (DZUB), from three distinct populations collected in three environmental protection areas of the Federal District, Brazil: Fazenda Água Limpa (FAL‐UnB) (*n* = 72); Brasília National Park (PNB) (*n* = 45); and Embrapa Cerrados (EC) (*n* = 38) (Figure S1). The three populations were chosen to improve our sampling effort, and to include more morphological variation. All measured individuals were collected from the same vegetation formation (typical savanna, locally known as “cerrado *sensu stricto*”), to avoid bias related to habitat variation. We selected more males than females to better capture the variation among them, as males exhibit three distinct morphotypes, while females do not. For body mass measurements, we weighed the dry individuals using a BEL Engineering M214Ai analytical balance (0.0001 g). Dry mass is a widely used measurement in entomology as a proxy for individual biomass. After weighing, we hydrated the individuals in hot water for 5 min and, using tweezers and a surgical scalpel, we removed the elytra, membranous wings, and the left anterior leg. The dissected structures were standardized and fixed with water‐based white glue on a strip of paper with a weight greater than 200 g/m^2^. Using a stereoscopic microscope, we individually photographed the pronotum, wings, elytra, protibia, and clypeus of each individual. With Image J software, we measured the pronotum width, horn length, and wing length (distance between landmarks 1 and 23—Figure 1B) and area of each individual twice, with the structure's measurement being the average of both measurements. We calculated the wingspan as 2 × wing length + pronotum width.

### 2.3 Digitization of Anatomical Landmarks

For the digitization of anatomical landmarks (Figure 1B) of the clypeus, protibia, elytra, and wing, we used TPSdig v. 2.32 software (Rohlf 2021). For the clypeus, we established 9 anatomical landmarks, marking the anterior margin of the clypeus (landmarks 4, 5, 6), the ends of the fronto‐clypeal suture (4 and 6), the eyes (1, 2, 8, and 9), and the paraocular regions (2, 3, 4, 6, 7, and 8). For the protibia, we established 11 anatomical landmarks, marking the tibial teeth (2, 3, 4, 5, 6, 7, 8, and 9) and 10 semi‐landmarks distributed along two curves to mark the posterior carina of the protibia and the anterior curvature of the protibia. For the elytra, we established 15 anatomical landmarks distributed between the beginning (9, 10, 11, 12, 13, 14, and 15) and the end (1, 2, 3, 4, 5, 6, 7) of the striae and 8 semi‐landmarks marking the lateral curvature of the elytra. For the wing, we established 23 anatomical landmarks, marking the radial (2, 3, 7, 9, 14, 15, 19), cubital (5, 10), medial (4, 6, 8), anal lobe (11, 12, 16), and jugal (16, 22, 23) veins.

### 2.4 Geometric Morphometric Variables

To obtain variables representing the shape of the structures (i.e., uniform components and partial warps) and visualize the shape, we used TPSrelW v. 1.75 software (Rohlf 2021), which uses Generalized Procrustes Analysis (GPA). GPA removes any information related to the position, orientation, and size of the structures from the coordinates of the anatomical landmarks (Bookstein 1997; Dryden and Mardia 1998). Before submitting the landmarks to GPA, the semi‐landmarks of the protibia and elytra were slid using TPSutil ver. 1.83 software (Rholf 2023) to minimize the arbitrary effects of their positions (Gunz and Mitteroecker 2013). Additionally, the size of each structure for all individuals was obtained from the centroid size (CS), defined as the square root of the sum of the squared distances between each landmark and the centroid of the configuration representing the shape (i.e., the mean of all coordinates) (Bookstein 1997). This allowed us to determine and, if necessary, remove the allometric effects of the structures in subsequent analyses.

### 2.5 Flight Biomechanics Indices

After collecting dry body mass, wing area, wingspan, and wing length, we calculated Wing Aspect Ratio (WAR—Wing length^2^/Wing area), Wing Loading (WL—Body weight/Wing area), and Wing Loading Moment (WLM—Body weight/Wingspan × Wing area). High WAR values indicate high‐speed and long‐distance flights, while low values indicate more maneuverable and slower flights (Dudley 2002). High WL values are associated with higher energy expenditure during flight, whereas lower values are associated with optimized flights with lower energy costs (Dudley 2002). High WLM values are related to higher wing beat frequencies, whereas lower values are associated with slower wing beat rates (Viscor and Fuster 1987).

### 2.6 Data Analysis

**FIGURE 2:**
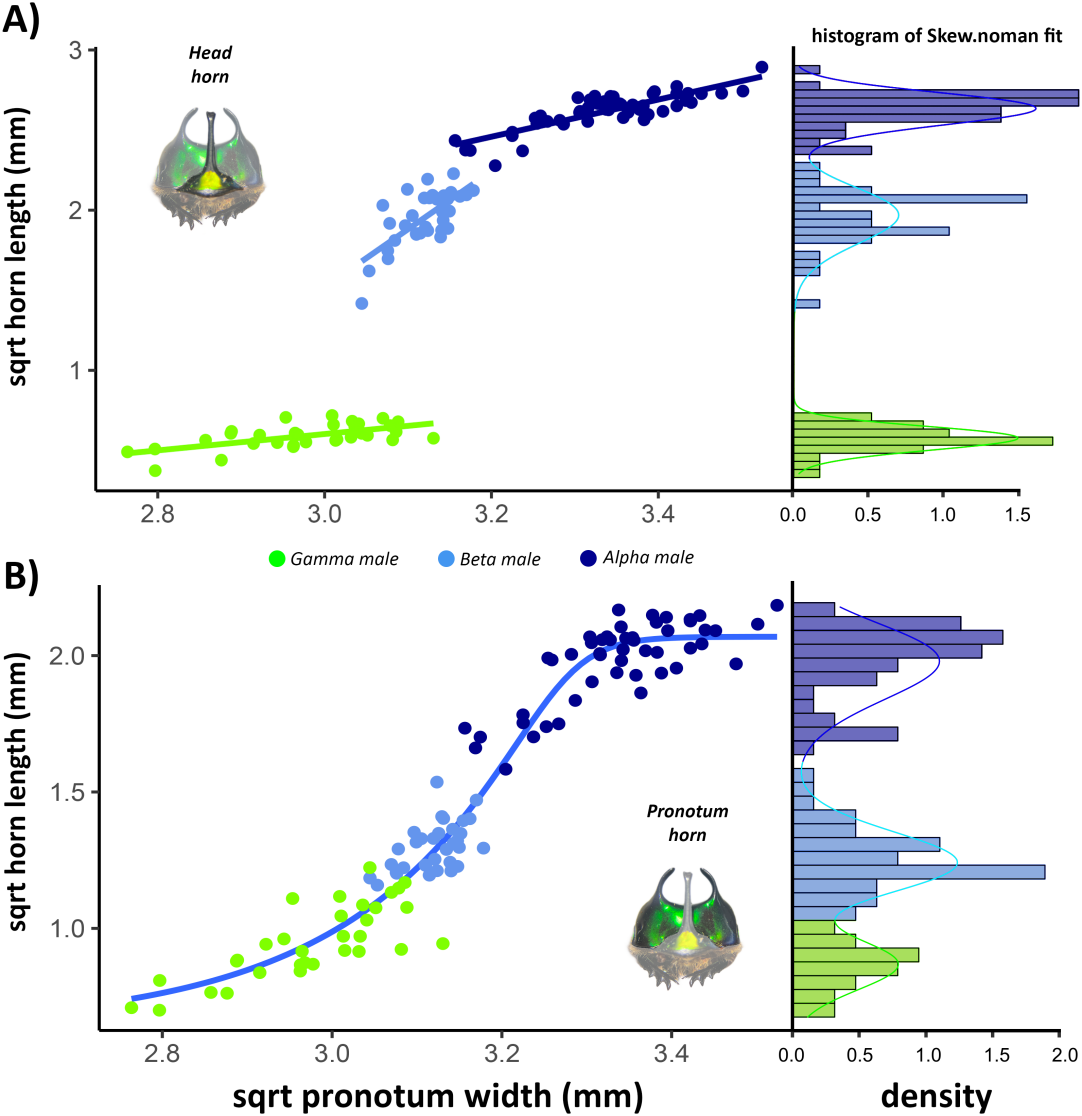
Regressions between the square root of head horn length (A) and pronotal horn length (B), and the square root of pronotal width, for trimorphic males of *Oxysternon palemo* (Scarabaeinae: Phanaeini). Head horn length is described by a linear regression, while pronotal horns are described by a five‐parameter logistic curve. On the right, a histogram showing the density and frequency of horn lengths, indicating trimodal patterns.

To classify the *O. palemo* males into each morphotype (alpha, beta, and gamma), we examined the distributions of head horn length values for trimodality using non‐parametric density curves (Rowland and Emlen 2009). In R software, using the mixsmsn package (Prates, Lachos, and Cabral 2013), we parameterized the distributions as mixtures of three skew‐normal distributions (Figure 2). Using the same package, we classified males into each morphotype based on their probability of occurrence (Table S1). We applied the same methodology to evaluate the distribution of pronotal horn length values, which also showed a trimodal distribution (Figure 2). For subsequent analyses, to better understand variations between groups, we classified females as one of the four levels along with the male morphotypes.

### 2.7 Morphometric Analysis

We then conducted Linear Discriminant Analysis (LDA) using morphotypes as groups based on the shape variables of the structures to obtain new axes summarizing the information in the original variable set. This ordination analysis was chosen to maximize segregation between groups according to the structure shapes. For each structure, we generated linear models between the first two axes of the discriminant analysis and the centroid size of each individual to assess potential allometric effects. In cases of significant (*p* < 0.05) and relevant (*r* ^2^ > 0.10) effects, we used the normalized residuals for subsequent analyses. This approach controls for allometric effects, allowing the evaluation of structure shapes independent of size (i.e., adaptive morphology) (Doube et al. 2011). After removing the allometric effect, we produced a plot to visualize the dispersion of individuals concerning the shape variation of each structure. For visualizing deformation and shape variation, we used TPSregr 1.43 software (Rohlf 2021), based on the individual scores from the discriminant analyses or normalized residuals when allometric effects were detected. To test for morphological differences between morphotypes, we used these values in a permutational multivariate analysis of variance (PERMANOVA), accompanied by post hoc tests to evaluate which groups differed from each other (*p* < 0.05).

### 2.8 Phenotypic Integration

Since the pronotal horns of *O. palemo* follow a clearly sigmoidal distribution, we used a five‐parameter logistic regression to adjust the allometric relationship of the square root of horn length (dependent variable) to the square root of pronotal width (independent variable) using the R package drc (Ritz et al. 2015). The morphological variables were square root‐transformed for a better model fit based on the inspection of residuals. Additionally, for the linear model concerning the head horn, we included the morphotype (alpha, beta, and gamma) as an interaction factor. From the generated models, we obtained the relative size of each horn as the residuals of each model, following a widely used methodology for dung beetles (Rowland et al. 2020).

To evaluate phenotypic integration between the horns and the shape of the structures, we tested covariations using linear models for each axis of the LDA separately after removing the allometric effect, in function of the relative size of each horn individually, with an interaction term for the morphotype factor. The significance of the models was assessed with an Analysis of Deviance (ANODEV) using the F‐test. To analyze the integration between the shape of the structures and the relative size of the head horn, we excluded gamma males from the analyses, as these individuals do not develop prominent horns.

### 2.9 Analysis of Biomechanical Variation

To assess the variation in flight biomechanics indices (response variables) between sexes and male morphotypes (predictor variables), we used Generalized Linear Mixed Models (GLMM), considering different populations as a random variable. Model selection was based on the Akaike Information Criterion (AIC) from all possible models, selecting the one with the lowest AIC, indicating the best fit (Johnson and Omland 2004). For the predictor variables selected in the best‐supported model, *p* values were obtained using likelihood ratio tests (Zuur et al. 2009). The significance of the models was assessed with an Analysis of Deviance (ANODEV) using the F‐test. Differences between variable levels were assessed through model contrast analyses. All models were performed using the R software (R Core Team 2016).

## 3 Results

### 3.1 Morphological Variations

**FIGURE 3:**
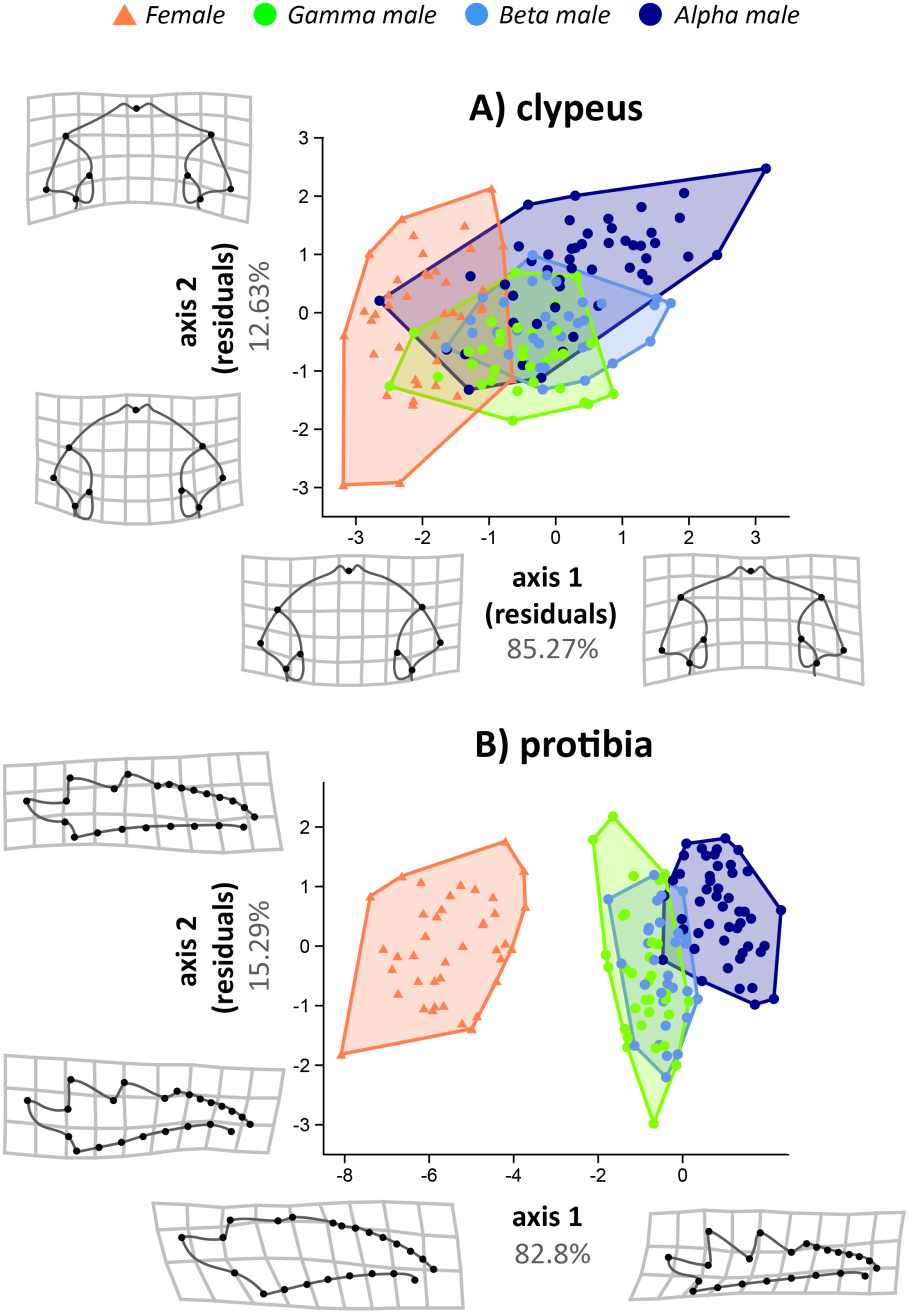
Principal axes of Linear Discriminant Analysis (LDA) regressed by centroid size, between females and trimorphic males (alpha, beta, gamma) of the dung beetle *Oxysternon palemo* (Scarabaeinae: Phanaeini). (A) Clypeus; (B) Protibia. For each structure, we generated linear models between the first two discriminant axes and the centroid size of each individual. For cases where there was a significant and relevant effect (R^2^ > 0.10, *p* < 0.05), normalized residuals were used to construct the plot. Grids indicate the direction of shape change in structures according to the scores obtained on the LDA axes or residuals between centroid size and LDA axes, when allometric effects were detected. Despite overlap between sexes and morphotypes in the morphospace of the main LDA axes, the shape of the clypeus (A) differed significantly between all groups (*p* < 0.001). Gamma and beta males did not significantly differ in protibia shape (B) (*p* > 0.05).

**FIGURE 4:**
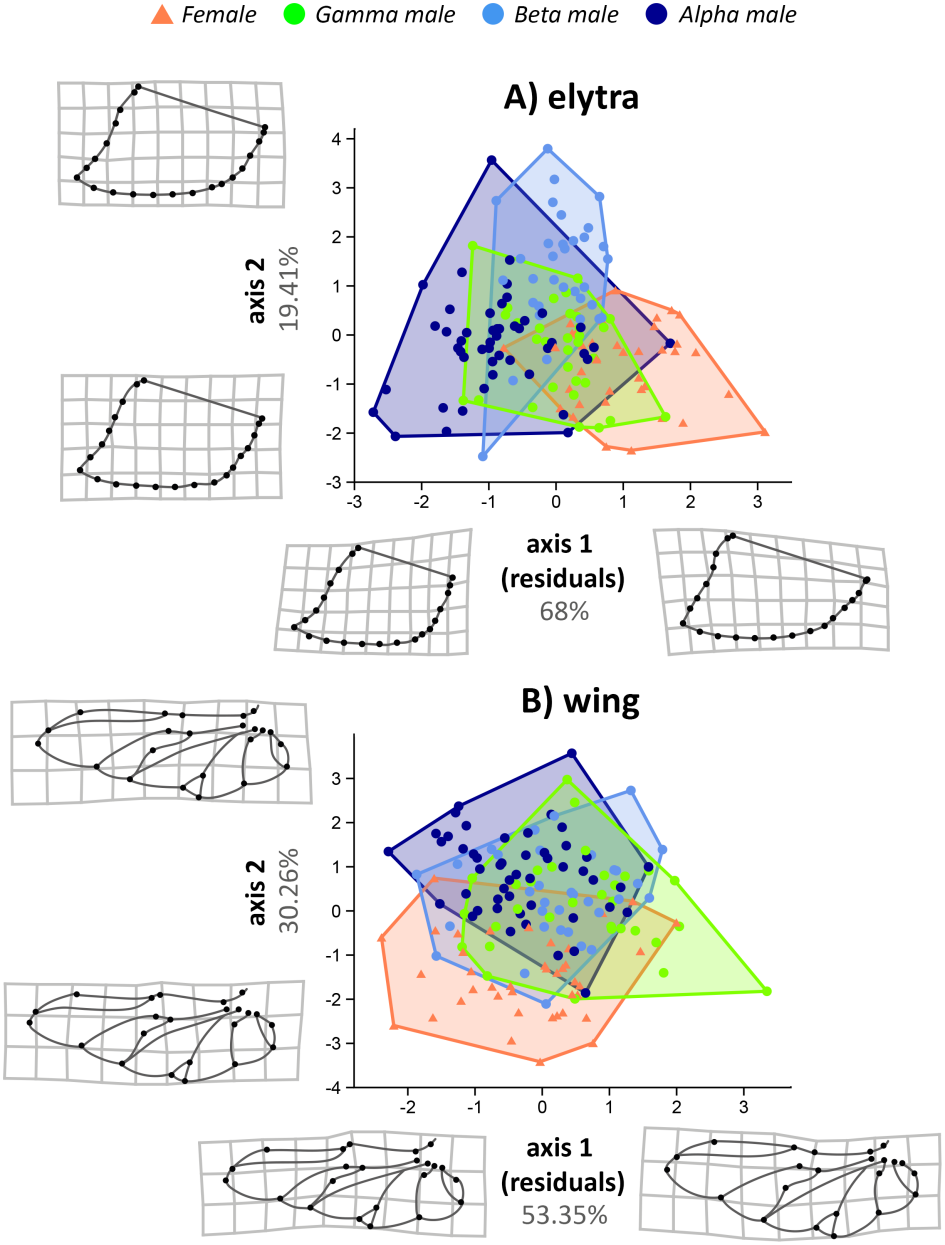
Principal axes of Linear Discriminant Analysis (LDA) regressed by centroid size, between females and trimorphic males (alpha, beta, gamma) of the dung beetle *Oxysternon palemo* (Scarabaeinae: Phanaeini). (A) Elytra; (B) Wing. For each structure, we generated linear models between the first two discriminant axes and the centroid size of each individual. For cases where there was a significant and relevant effect (R^2^ > 0.10, *p* < 0.05), normalized residuals were used to construct the plot. Grids indicate the direction of shape change in structures according to the scores obtained on the LDA axes or residuals between centroid size and LDA axes, when allometric effects were detected. Despite overlap between sexes and morphotypes in the morphospace of the main LDA axes, the shape of the elytra (A) differed significantly between all groups (*p* < 0.001). For the wing (B), only beta and gamma males did not differ significantly (*p* = 0.18).

The two canonical axes resulting from the discriminant analyses explained 97.9% of the original data variation for the clypeus (Figure 3A), 98.09% for the protibia (Figure 3B), 87.41% for the elytra (Figure 4A), and 83.61% for the wing (Figure 4B). Allometric effects were detected for axis 1 (*t* = 13.67, *r* ^2^ = 0.78, *p* = 0.0001) and axis 2 (*t* = −9.28, *r* ^2^ = 0.36, *p* = 0.0001) of the clypeus, though this effect was significant for males only on axis 1 (Figure S2a). For the protibia, allometry was detected on axis 2 (*t* = 13.41, *r* ^2^ = 0.54, *p* = 0.0001), and similarly to the clypeus, the effect on axis 1 was significant for males only (*t* = 4.01, *r* ^2^ = 0.12, *p* = 0.0003) (Figure S2c). For the elytra, allometric effects were detected on axis 1 (*t* = −8.58, *r* ^2^ = 0.32, *p* = 0.0001) but not on axis 2 (*t* = −1.32, *r* ^2^ = 0.01, *p* = 0.17). Similarly, for the wing, allometric effects were detected on axis 1 (*t* = −13.988, *r* ^2^ = 0.56, *p* = 0.0001) but not on axis 2 (*t* = −0.13, *r* ^2^ = 0.0001, *p* = 0.9). Comparisons of morphological differences between morphotypes indicated significant differences in all structures across at least three of the four studied groups (females and alpha, beta, and gamma males) (Figures 3 and 4). For the clypeus shape, we found significant adaptive differences between all groups (PERMANOVA; *F*_3,151_ = 40.94, *p* = 0.001) as indicated by post hoc tests (Table S2). Alpha males tend to have a shortened and flattened anterior margin of the clypeus, whereas females and gamma males tend to have a more elongated and narrow anterior margin. Additionally, the margins near the fronto‐clypeal suture are more pronounced in alpha males but shortened in gamma males and females, resulting in a “shovel” shape (Figure 3A). For the protibia (*F*_3,147_ = 311.85, *p* = 0.001), we found significant differences between all groups except gamma and beta males (Table S2), as well as pronounced sexual dimorphism. Females have broader protibia with shortened tibial teeth, whereas males have narrower protibia with pronounced tibial teeth. Moreover, alpha males have a straighter distal portion of the protibia, whereas gamma and beta males have a more arched proximal region (Figure 3B). For the elytra (*F*_3,149_ = 3.35, *p* = 0.001), significant differences were found between all groups (Table S2), with males having a shorter caudal region and females having elongated elytra (Figure 4A). For the membranous wings (*F*_3,151_ = 25.45, *p* = 0.001), significant differences were found between all groups except beta and gamma males (Table S2). Gamma males have elongated posterior regions (landmarks 1, 4, 5, 11, 12, and 16) and shortened jugal lobes (landmarks 16, 22, and 23), whereas alpha males and females have shortened posterior regions and widened jugal lobes. Thus, gamma males tend to have an upward arching wing shape, whereas alpha males and females tend to have a tapering distal wing. Additionally, females and alpha males have expanded and developed radial and medial innervation (landmarks 7, 8, 9, 10, 14, and 15), compared to the narrower corresponding region in gamma males' wings (Figure 4B).

### 3.2 Phenotypic Integration

**FIGURE 5:**
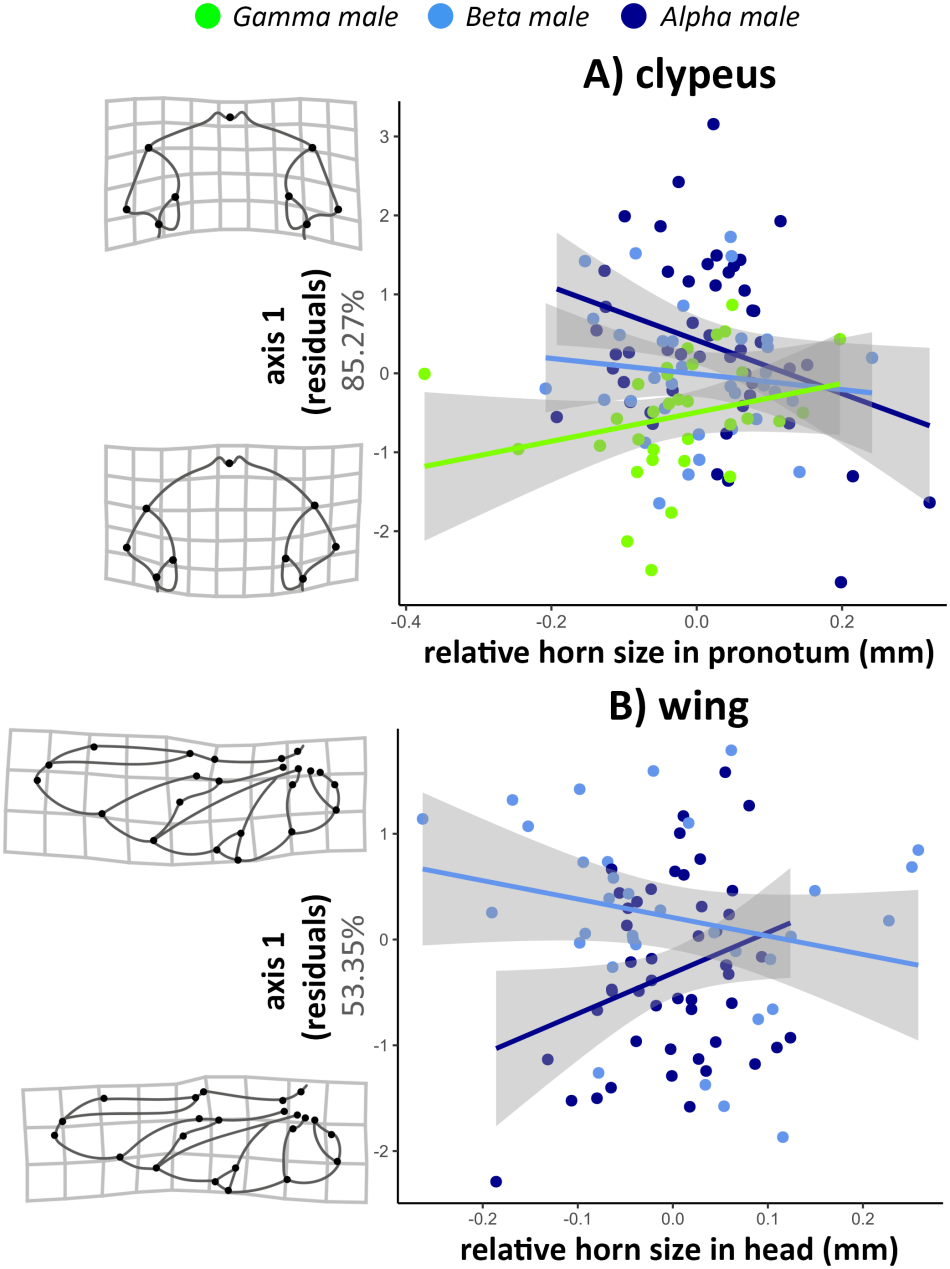
Phenotypic integration (i.e., covariation) between (A) residual shape (without allometric effect) of the clypeus and relative size of the pronotal horn, and (B) residual shape (without allometric effect) of the wing and relative size of the head horn in trimorphic males (alpha, beta, gamma) of *Oxysternon palemo* (Scarabaeinae: Phanaeini).

When evaluating potential phenotypic integrations between the relative horn sizes in males and the shape of the analyzed structures, we found covariations only for the clypeus and wings (Table 1). For the clypeus, there were distinct covariations between its shape (without allometric effect) and the relative size of pronotal horns for each male morphotype. Our results indicated that the larger the relative size of the horns, the longer and more rounded the clypeus in alpha males. Conversely, the smaller the horn size, the shorter and more angular the clypeus. For gamma males, this correlation was reversed (Figure 5A). Similarly, for the membranous wings, there was covariation between their shape (without allometric effect) and the relative size of the head horn. The larger the relative horn size, the more expanded the distal portion of the wing in alpha males. However, individuals with smaller horns had more tapered wings. For beta males, this correlation was reversed (Figure 5B).

**TABLE 1:** Covariation between the relative length (i.e., residual) of the head horn (RHH) and pronotal horn (RHP), and the residual shape of the clypeus, protibia, wing, and elytra (i.e., axes of Discriminant Analysis regressed by centroid size).

Variables	(a) Axis 1 of clypeus shape					(b) Axis 2 of clypeus shape				
	df	SS	MS	*F*	*p*	df	SS	MS	*F*	*p*
RHH	1	0.07	0.06	0.06	0.79	1	1.76	1.76	3.27	0.07
Morph	1	2.83	2.82	2.80	0.09	1	19.38	19.38	35.95	0.001
RHH × morph	1	0.02	0.02	0.002	0.96	1	0.21	0.21	0.40	0.75
Residuals	79	79.83	1.01			79	42.61	0.53		
RHP	1	0.223	0.223	0.26	0.60	1	4.23	4.23	5.12	0.02
Morph	1	14.097	14.097	16.73	< 0.001	1	19.81	19.81	23.95	< 0.001
RHP × morph	1	6.19	6.19	7.35	0.007	1	0.51	0.51	0.62	0.43
Residuals	111	93.48	0.84			111	91.79	0.82		

Variables	(c) Axis 1 of tibia shape					(d) Axis 2 of tibia shape				
	df	SS	MS	*F*	*p*	df	SS	MS	*F*	*p*
RHH	1	0.43	1.20	1.21	0.27	1	0.004	0.004	0.005	0.94
Morph	1	34.76	66.49	96.22	< 0.001	1	17.81	17.81	25.79	< 0.001
RHH × morph	1	0.01	0.26	0.03	0.84	1	0.01	0.01	0.01	0.90
Residuals	75	27.09	0.36			75	51.81	0.69		
RHP	1	1.20	1.20	3.06	0.08	1	0.19	0.19	0.22	0.63
Morph	1	66.49	66.49	169.65	< 0.001	1	26.18	26.18	29.53	< 0.001
RHP × morph	1	0.26	0.26	0.68	0.41	1	0.61	0.61	0.69	0.40
Residuals	107	41.93	0.39			107	94.88	0.88		

Variables	(e) Axis 1 of elytra shape					(f) Axis 2 of elytra shape				
	df	SS	MS	*F*	*p*	df	SS	MS	*F*	*p*
RHH	1	0.18	0.18	0.34	0.55	1	0.51		0.41	0.49
Morph	1	16.59	16.59	31.71	< 0.001	1	41.41		33.74	< 0.001
RHH × morph	1	0.00	0.0002	0.0004	0.98	1	0.07		0.06	0.75
Residuals	77	40.30	0.52			77	94.5	1.22		
RHP	1	1.23	1.23	2.32	0.13	1	0.13	0.13	0.08	0.02
Morph	1	17.65	17.65	33.16	< 0.001	1	8.93	8.93	5.83	0.01
RHP × morph	1	0.35	0.35	0.62	0.42	1	0.37	0.37	0.24	0.43
Residuals	109	58.02	0.53			109	166.95	1.53		

Variables	(g) Axis 1 of wing shape					(h) Axis 2 of wing shape				
	df	SS	MS	*F*	*p*	df	SS	MS	*F*	*p*
RHH	1	0.02	0.02	0.03	0.84	1	0.02	0.02	0.02	0.87
Morph	1	5.53	5.53	7.90	0.006	1	3.60	3.60	3.57	0.06
RHH × morph	1	4.33	4.33	6.18	0.01	1	0.94	0.94	0.93	0.33
Residuals	79	55.35	0.70			79	79.59	1.00		
RHP	1	0.71	0.71	0.47	0.49	1	0.02	0.02	0.02	0.87
Morph	1	10.55	10.55	11.96	< 0.001	1	3.60	3.60	3.57	0.06
RHP × morph	1	2.26	2.26	2.56	0.11	1	0.94	0.94	0.93	0.33
Residuals	111	97.95	0.88			111	79.59	1.00		

### 3.3 Biomechanical Variations

**FIGURE 6:**
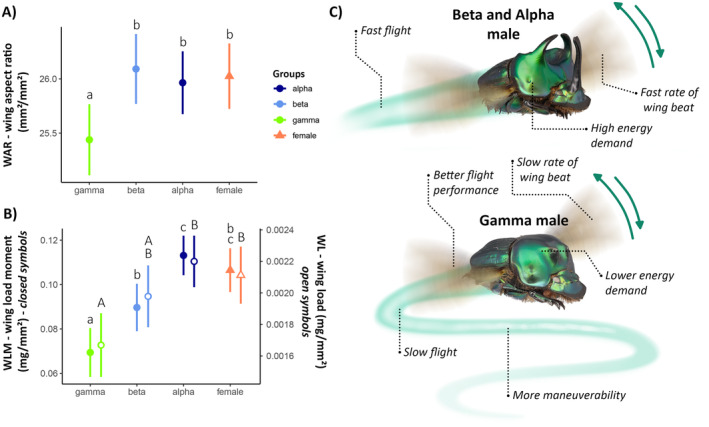
Mean variation of wing aspect ratio (WAR) (A); wing loading moment (WLM) (closed forms) and wing loading (WL) (open forms) (B) among females (triangles) and trimorphic male morphotypes: Alpha (dark blue), beta (light blue), and gamma (green) of the dung beetle *Oxysternon palemo* (Scarabaeinae: Phanaeini). Wing Aspect Ratio (WAR) was calculated as Wing Length^2^/Total Wing Area; Wing Loading (WL) as Body Weight/Wing Area; and Wing Loading Moment (WLM) as Body Weight/Wing Span × Wing Area. (C) Gamma males exhibit lower values of WAR (*p* < 0.001), WL (*p* < 0.001), WLM (*p* < 0.01), indicating they have slower flight, lower energy expenditure, and greater maneuverability compared to the other groups. Different letters between groups indicate significant differences (*p* < 0.05) according to Tukey's post hoc test.

When evaluating the differences in flight biomechanics indices between the groups, we found that wing aspect ratio (WAR) differed significantly between the groups (*F*_3,149_ = 6.5, *p* < 0.0001), with gamma males having shorter and broader wings (lower WAR values), whereas the other groups did not differ from each other but had more slender and elongated wings (higher WAR values; Figure 6A and Table S3). For wing loading (WL; *F*_3,149_ = 5.89, *p* < 0.0001), significant differences were found between the groups, with gamma males having lower values than alpha males and females, indicating more compensatory flight and lower energy expenditure (Figure 6c). Beta males did not differ from females and gamma and alpha males in WL (Figure 6B and Table S3). For wing loading moment (WLM; *F*_3,149_ = 14.23, *p* < 0.0001), significant differences were found between the groups, with gamma males having lower values than beta, alpha males, and females, indicating lower wingbeat frequencies (Figure 6C). Females did not differ from beta and alpha males in WLM (Figure 6B; Table S3).

## 4 Discussion

Our results reveal that both functionally important morphological structures for dung beetles and flight biomechanics factors can vary plastically between sexes and among male morphotypes of *O. palemo*. Additionally, we found covariations between the relative horn sizes in males and the shapes of these structures. This variation and covariation are likely adaptive responses to different selective pressures, resulting from behavioral and niche differences between sexes and among male morphotypes. Thus, through phenotypic plasticity, the analyzed morphological structures might respond to ecological differences linked to sex and morphotypes of the species, providing an adaptive value for their functions in the environment.

### 4.1 Morphological Variations

The functionality of digging structures, such as the clypeus and protibia, is directly linked to the processes of nest construction and provisioning (Scholtz, Davis, and Kryger 2009; Linz, Hu, and Moczek 2019) and communication among conspecifics (Ribeiro et al. 2024). In Phanaeini, during these processes, bisexual cooperation is widely documented and increases reproductive success (Halffter and López‐Guerrero 1977; Rasmussen 1994). Thus, the differences found in the digging structures may be plastic adaptive responses selected for the performance of these functions. It is known that the tibial teeth increases digging speed and aids in nesting (Linz, Hu, and Moczek 2019), but it is also possible that features such as protibial width assist in these processes, although this has not yet been tested. Moreover, the tibial teeth are also correlated with resource partitioning and fragmentation (Edmonds 1972; Halffter and Edmonds 1982). We therefore suggest that the elongated shape of the clypeus and robust, more blunt protibia in females are likely directly associated with tunnel excavation, while the shortened clypeus and pronounced tibial teeth of the slimmer male protibia may be more efficient for resource partitioning and allocation.

Moreover, we observed distinct variations in the arching of the protibia among males, where alpha males have a less arched distal region. Similar patterns have been observed in the dung beetle *Onthophagus taurus*, where less arched protibia in males with relatively larger horns optimize their support in tunnels during physical combats (Rohner, Macagno, and Moczek 2020). The different reproductive strategies adopted by polyphenic males of dung beetles indicate that hornless males have greater mobility within tunnels (Cummings, Evans, and Chaves‐Campos 2018; Madewell and Moczek 2006) and excavate adjacent tunnels (Emlen 1997; Moczek and Emlen 2000). Therefore, we suggest that greater excavation and locomotion capabilities, along with stealthy behavior within tunnels, may have selected for clypeus and protibia shapes in gamma males more similar to those of females.

For diurnal Phanaeini, including *O. palemo*, sexual dichromatism related to clypeus coloration has been documented, possibly associated with the identification and communication of conspecifics (Ribeiro et al. 2024). It remains unclear how sexual selection influences clypeus coloration in males (Ribeiro et al. 2024), but it is possible that the shape variation found in clypeus could influence these signaling patterns.

In male horned dung beetles, sexual selection is directly linked to disputes in agonistic encounters (Rasmussen 1994). Given the intense competition resulting from these encounters (Moczek and Emlen 2000), the morphometry of the elytra suggests that the robust body and short abdomen of alpha males are associated with investment in pronotal musculature (i.e., greater investment in combat and dispersal than in reproduction) (Knell and Parrett 2023; Simmons, Tomkins, and Hunt 1999; Simmons, Emlen, and Tomkins 2007; Simmons and Emlen 2008). Similarly, the same pattern of reproductive investment explains the elongated elytra and abdomen in females (Raine et al. 2018; Srygley and Chai 1990).

Reproductive success in dung beetles directly depends on their dispersal capability, facilitated by the sclerotization of the membranous wing veins, which prevents wing collapse during flight (Scholtz, Davis, and Kryger 2009). Hence, we posit that the enlarged radial and cubital veins in alpha males and females reflect increased wing loading (WL) pressure and the associated wing loading moment (WLM). Conversely, despite minimal environmental selective pressure on dung beetle wing shapes (Bai et al. 2011; Ospina‐Garcés et al. 2018), anal and jugal wing regions are more susceptible to change (Bai et al. 2011), linked to the prolonged, swift flight patterns of diurnal species (Tocco, Dacke, and Byrne 2019). Thus, the trend of increased jugal area in female and alpha male wings, compared to the reduction in gamma male wings, directly relates to differing flight speeds, foraging strategies, and dispersal capabilities (Ospina‐Garcés et al. 2018; Tocco, Dacke, and Byrne 2019). Additionally, longer, narrower wings in alpha males may compensate for aerodynamically costly horns (Hunt, Kotiaho, and Tomkins 1999).

### 4.2 Phenotypic Integration

Sexual selection is expected to modify secondary sexual characteristics in a manner that compensates for fitness costs (Hosken and House 2011). Our analysis suggests that the different horns of *O. palemo* may be phenotypically integrated through covariation with distinct structures. Unlike findings in dung beetle *O. taurus* (Rowland et al. 2020), we found no covariation between *O. palemo* horns and protibia shape. However, we observed covariation between pronotal horns and clypeus shape, and between head horns and wing shape. These findings reinforce the notion that different horns within an organism serve different functions under distinct evolutionary pressures (Emlen 2001).

Within an evolutionary context, intense resource competition may favor intraspecific signaling to reduce aggressive interactions, allowing individuals to avoid conflicts with stronger or more motivated competitors (Searcy and Nowicki 2010; Smith and Harper 2003). Moreover, intraspecific communication during reproductive contexts may favor colors and patterns that enhance detectability and recognition (Endler 1984). Given the sexual dichromatism in the clypeus of diurnal Phanaeini, where males exhibit pronounced iridescent coloration in this region (Ribeiro et al. 2024), we hypothesize that covariation between relative horn size on the pronotum and clypeus shape (Figure 5A) could signal competitive ability status among individuals. Thus, in the context of high competition among alpha males, individuals with relatively larger horns and elongated clypeus could signal their competitive ability to potential rivals (i.e., other males) through color signals (e.g., area, shape, or reflectance of colored spot). Similarly, in other insects like paper wasps (Hymenoptera: Vespidae: *Polistes*), the number of black spots on the clypeus acts as an indicator of status, mediating conflicts over feeding and reproduction among colony females (Tibbetts 2002). Furthermore, Phanaeini beetles, like *O. palemo*, have sufficient visual acuity to use the clypeus for short‐distance color vision communications (Théry, Pincebourde, and Feer 2008; Vulinec 1997). Under the premise of honest signaling of combat ability status, smaller males are expected to show an inverse correlation, which would explain the observed pattern; however, this has not yet been tested. Future research should assess whether variation in male clypeus shape alters brightness and chroma‐visual values and whether these signals encode information about male quality.

Unlike other visually selected sexual ornaments (e.g., flashy feathers in birds), beetle horns are dense and robust adaptations to selection pressures for combat (Emlen 1997). Unlike visual ornaments, horn length exerts little to no negative effect on aerodynamics during male beetle flight (Hongo 2010; McCullough and Tobalske 2013), but horns can incur high energy costs due to their weight (Goyens et al. 2015). From a foraging and locomotion standpoint, the production of these armaments and sexual ornaments is generally offset by compensatory characteristics (e.g., wing modifications), reducing aerodynamic (Evans and Thomas 1992) and energy costs (Goyens et al. 2015). Thus, the positive correlation found between relative head horn size and widening of the distal region of membranous wings in males with large horns (i.e., alpha) may compensate for increased wing loading. Conversely, for males with small horns (i.e., beta males), the smaller horn size may not significantly affect wing load, making the investment in wings for fast flight less costly. Surprisingly, a similar pattern of covariation between membranous wings and horns in larger and smaller males was found in the dung beetle *O. taurus* (Rohner, Macagno, and Moczek 2020). Therefore, elaborate morphologies in alpha males may be compensated by wing morphology adjustments, whereas less exaggerated morphologies in beta males may not necessarily require such compensations.

### 4.3 Biomechanical Variations

In beetles, wing loading and individual size positively correlate with dispersal capability and flight speed (Gibb et al. 2006; Hongo 2010; Shegelski, Evenden, and Sperling 2019). Gamma males consistently exhibited lower values for all flight biomechanics factors, suggesting reduced dispersal capability (Berwaerts, Van Dyck, and Aerts 2002; Dudley 2002) and slower flight speeds (Hongo 2010). Physiologically, lower WL and WLM values in gamma males also represent lower energy costs and optimized flight (Dudley 2002; Gibb et al. 2006; Martínez‐Pérez, Galante, and Micó 2022), with lower WAR values indicating increased maneuverability (Dudley 2002). However, the studies on insect flight performance indicate that speed and flight pattern also depend on thoracic muscle mass (McCullough and Emlen 2013), thermoregulatory capacity (Chapman 1998), and ambient temperature (Danthanarayana 1986). Therefore, although wing morphological variations and flight biomechanics indicate a consistent trend, other variables or secondary characteristics may influence species foraging patterns.

Insects often face trade‐offs between reproductive investment and dispersal (Helms and Kaspari 2015; Zera and Denno 1997). Under the perspective of intense sperm competition in polyphenic dung beetles (Kelly 2008; Simmons, Tomkins, and Hunt 1999; Simmons and Emlen 2008), enhancing fitness associated with alpha male guarding behavior requires natural selection to favor alpha males that locate and mate with females quickly. Conversely, enhancing fitness associated with sneaky behavior in gamma males requires natural selection to favor sperm competition (Knell and Parrett 2023). Thus, we hypothesize that selective pressures on different male morphs are distinct; for alpha males, swift, long‐distance flight ensures first arrival at resources, preventing copulation by other males with females, whereas in gamma males, slower, shorter flights with lower energy expenditure lead to delayed resource arrival, but favor investment in reproductive competition, adopting stealthy secondary tactics for reproduction. Similar patterns supporting this hypothesis were found in the dimorphic beetle *Trypoxylus dichotomus*, where larger males (alpha) exhibited superior average flight speeds compared to smaller males (beta and gamma) (Hongo 2010). For the dimorphic dung beetle *O. taurus*, larger males colonized resource sources more rapidly than smaller males (Hunt, Kotiaho, and Tomkins 1999). Similarly, in the trimorphic beetle *Librodor japonicus*, different male morphs invested in armament (alpha), dispersal speed, and reproductive capacity (beta and gamma) (Okada et al. 2008). Thus, our findings regarding wing morphometrics and flight biomechanics converge on distinct foraging patterns mediated by reproductive compensations.

Our results demonstrate how functionally important structures and biomechanical traits can vary between sexes and among polyphenic males under an adaptive perspective, reflecting alternative reproductive strategies and behaviors. Thus, we confirm our hypothesis that fossorial structures in smaller males and females are morphologically more similar due to selective pressures associated with tunnel excavation and mobility, whereas in alpha males, the shape of these structures appears to optimize combat and resource partitioning. Additionally, we found covariations between relative horn size and residual shape of the clypeus and wings, suggesting that these correlations may optimize visual communication through color signals and compensate for energy costs associated with the weight of armaments. In addition to these findings, we also support the hypothesis that wing morphology, along with flight biomechanics indices, describes distinct dispersal patterns among morphotypes. Therefore, our findings indicate that alpha and beta males invest more heavily in in‐flight intensity, whereas gamma males exhibit optimized flights with lower energy expenditure. Although our results on flight biomechanics and wing morphology are consistent with literature describing different foraging patterns mediated by reproductive investment, further studies quantifying the effects of these investment strategies (e.g., dispersal area) in dung beetles are needed.

## Author Contributions

**Pedro Henrique de Oliveira Ribeiro:** conceptualization (lead), data curation (lead), formal analysis (lead), investigation (lead), methodology (equal), project administration (equal), software (equal), supervision (supporting), validation (equal), visualization (lead), writing – original draft (lead), writing – review and editing (equal). **Nicholas Ferreira Camargo:** data curation (supporting), formal analysis (equal), investigation (supporting), methodology (equal), software (equal), validation (equal), visualization (supporting), writing – original draft (supporting), writing – review and editing (equal). **Marina Regina Frizzas:** conceptualization (supporting), funding acquisition (lead), investigation (supporting), project administration (equal), resources (lead), supervision (equal), writing – original draft (supporting), writing – review and editing (equal).

## Conflicts of Interest

The authors declare no conflicts of interest.

## Supporting information


**Figure S1.** Distribution map of occurrence records of the dung beetle *Oxysternon palemo* (Scarabaeinae: Phanaeini) in Brazil (adapted from Maldaner, Costa‐Silva, and Vaz‐de‐Mello 2024) and environmental protection areas of three distinct populations in the Federal District, Brazil, for specimen collection: Fazenda Água Limpa (FAL‐UnB), Brasília National Park (PNB), and Embrapa Cerrados (EC). Green area indicates Cerrado biome. Light green dots indicate points where the species occurs.

**Figure S2.** Regression between centroid size and discriminant analysis axes (LDA) for clypeus (a, b) and protibia (c, d), for males (a, c) and females (b, d) of *Oxysternon palemo* (Scarabaeinae: Phanaeini). For the clypeus and protibia, there was a significant correlation between centroid size and LDA axis 1 in males, but not for females.

**Table S1.** Classification of *Oxysternon palemo* (Scarabaeinae: Phanaeini) males into alpha, beta, and gamma morphotypes or alpha and beta morphotypes is based on the distributions of head horn length values using non‐parametric density curves (Rowland and Emlen 2009), implemented through the mixsmsn package (Prates, Lachos, and Cabral 2013) in R software.
**Table S2:** Post hoc pairwise PERMANOVA testing for body structures (clypeus, protibia, elytra, and wing) between females and trimorphic males (alpha, beta, and gamma) of *Oxysternon palemo* (Scarabaeinae: Phanaeini).
**Table S3:** Post hoc Tukey test between females and trimorphic males (alpha, beta, gamma) for mixed‐effects linear models (GLMM) of biomechanical indices: Wing Aspect Ratio (WAR), Wing Loading (WL), and Wing Loading Moment (WLM).
**Table S4:** All data. id (individual identification number); pop (individual’s population of origin); sex (sex); morphotype (alpha, beta, gamma, female); pro_larg (pronotum width); horn1 (horn length on head); horn2 (horn length on pronotum); war (wing aspect ratio); wl (wing loading); wlm (wing loading moment); resivc1norm_clypeus (residual clypeus shape); resivc2norm_clypeus (residual clypeus shape); axie1_pro (protibia shape); residvc2norm_pro (residual protibia shape); residvc1norm_eli (residual elytra shape); axis2_eli (elytra shape); resivc1norm_asa (residual wing shape); axis2_asa (wing shape).

## Data Availability

All data underlying this study are in the Supporting information.
