# Supplementary figures and images for: Adaptative Variation in a Neotropical Dung Beetle: Females and Gamma Males Present Tunneler Morphology, While Beta and Alpha Males Present Wing Morphology for Velocity
Source: Ecol Evol. 2024 Nov 24;14(11):e70457. doi: 10.1002/ece3.70457 (PMC11586238; doi:10.1002/ece3.70457)

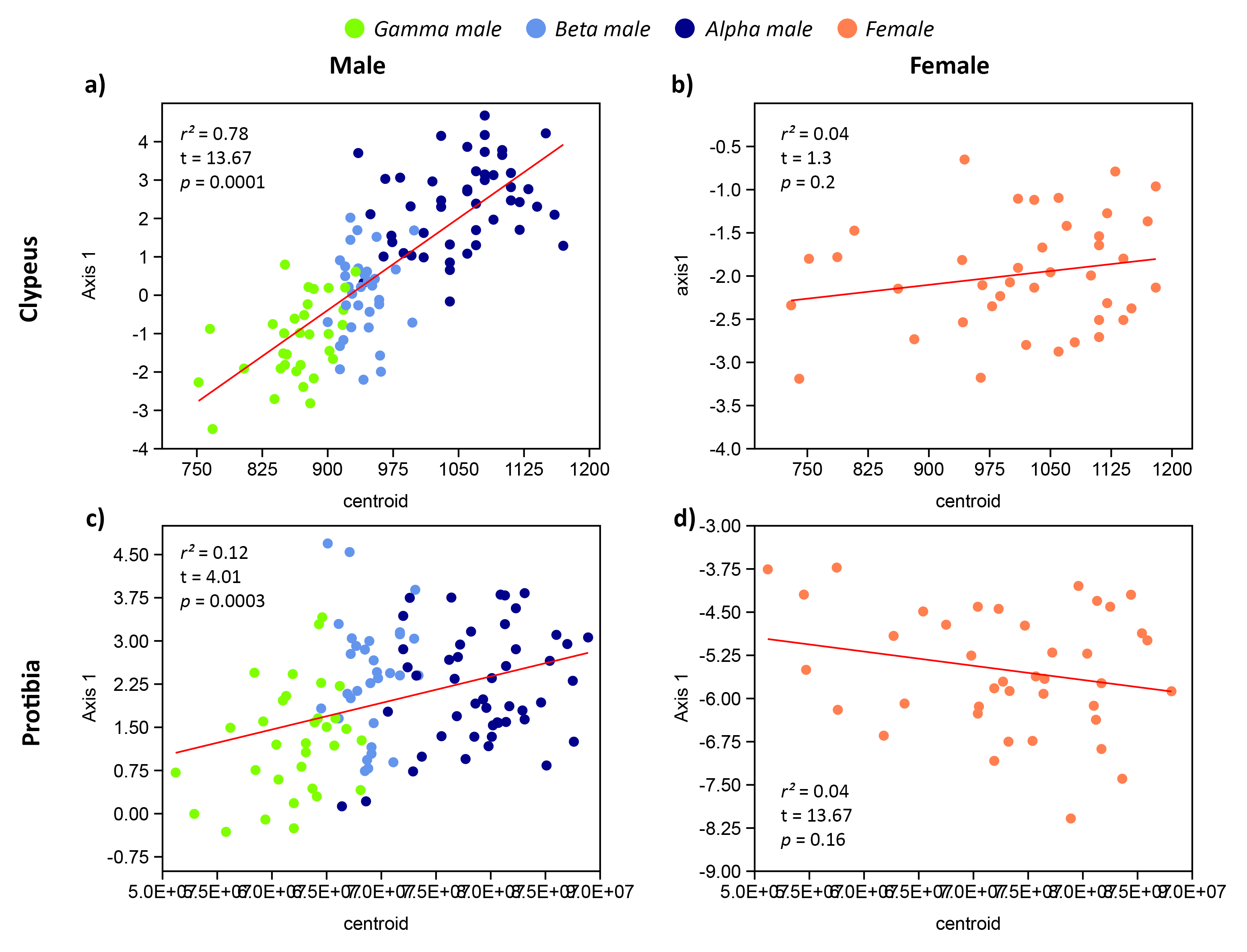

Supplement: Supplementary file 1 — Figure S1. Distribution map of occurrence records of the dung beetle Oxysternon palemo (Scarabaeinae: Phanaeini) in Brazil (adapted from Maldaner, Costa‐Silva, and Vaz‐de‐Mello 2024) and environmental protection areas of three distinct populations in the Federal District, Brazil, for specimen collection: Fazenda Água Limpa (FAL‐UnB), Brasília National Park (PNB), and Embrapa Cerrados (EC). Green area indicates Cerrado biome. Light green dots indicate points where the species occurs. [file ECE3-14-e70457-s003.tif]

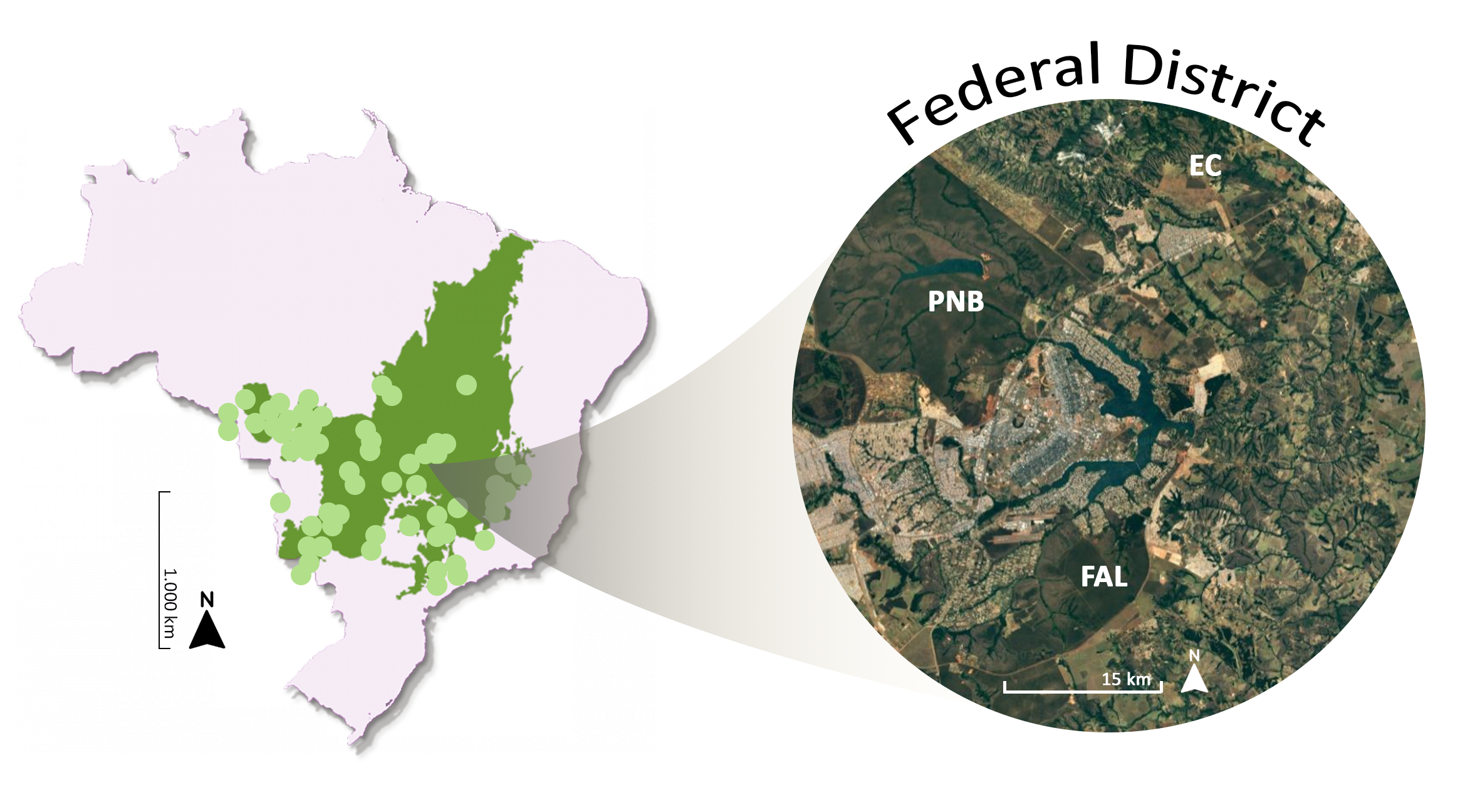

Supplement: Supplementary file 2 — Figure S2. Regression between centroid size and discriminant analysis axes (LDA) for clypeus (a, b) and protibia (c, d), for males (a, c) and females (b, d) of Oxysternon palemo (Scarabaeinae: Phanaeini). For the clypeus and protibia, there was a significant correlation between centroid size and LDA axis 1 in males, but not for females. [file ECE3-14-e70457-s002.tif]
