# Supplementary material for: Adaptative Variation in a Neotropical Dung Beetle: Females and Gamma Males Present Tunneler Morphology, While Beta and Alpha Males Present Wing Morphology for Velocity
Source: Ecol Evol. 2024 Nov 24;14(11):e70457. doi: 10.1002/ece3.70457 (PMC11586238; doi:10.1002/ece3.70457)
Supplement: Supplementary file 3 — Table S1. Classification of Oxysternon palemo (Scarabaeinae: Phanaeini) males into alpha, beta, and gamma morphotypes or alpha and beta morphotypes is based on the distributions of head horn length values using non‐parametric density curves (Rowland and Emlen 2009), implemented through the mixsmsn package (Prates, Lachos, and Cabral 2013) in R software. Table S2: Post hoc pairwise PERMANOVA testing for body structures (clypeus, protibia, elytra, and wing) between females and trimorphic males (alpha, beta, and gamma) of Oxysternon palemo (Scarabaeinae: Phanaeini). Table S3: Post hoc Tukey test between females and trimorphic males (alpha, beta, gamma) for mixed‐effects linear models (GLMM) of biomechanical indices: Wing Aspect Ratio (WAR), Wing Loading (WL), and Wing Loading Moment (WLM). Table S4: All data. id (individual identification number); pop (individual’s population of origin); sex (sex); morphotype (alpha, beta, gamma, female); pro_larg (pronotum width); horn1 (horn length on head); horn2 (horn length on pronotum); war (wing aspect ratio); wl (wing loading); wlm (wing loading moment); resivc1norm_clypeus (residual clypeus shape); resivc2norm_clypeus (residual clypeus shape); axie1_pro (protibia shape); residvc2norm_pro (residual protibia shape); residvc1norm_eli (residual elytra shape); axis2_eli (elytra shape); resivc1norm_asa (residual wing shape); axis2_asa (wing shape). [file ECE3-14-e70457-s001.zip › Supplmentary_tables.docx]

**Supplementary table 1:** Classification of *Oxysternon palemo* (Scarabaeinae: Phanaeini) males into alpha, beta, and gamma morphotypes or alpha and beta morphotypes is based on the distributions of head horn length values using non-parametric density curves (Rowland & Emlen, 2009), implemented through the mixsmsn package (Prates et al., 2013) in R software.

| **individuals** | **head horn** | | |
| --- | --- | --- | --- |
|  | **beta** | **gamma** | **alpha** |
| 1 | 0.548523619 | 0 | 0.451476381 |
| 2 | 1.1341E-06 | 0 | 0.999998866 |
| 3 | 0.000210282 | 0 | 0.999789718 |
| 4 | 6.02E-08 | 0 | 0.99999994 |
| 5 | 0.997913746 | 0 | 0.002086254 |
| 6 | 1.829E-07 | 0 | 0.999999817 |
| 7 | 0.999679067 | 0 | 0.000320933 |
| 8 | 3.502E-07 | 0.999999 | 6.541E-07 |
| 9 | 5.89E-08 | 0 | 0.999999941 |
| 10 | 8.88E-08 | 0 | 0.999999911 |
| 11 | 0.999999588 | 0 | 4.121E-07 |
| 12 | 5.302E-07 | 0 | 0.99999947 |
| 13 | 0.901903516 | 0 | 0.098096484 |
| 14 | 3.237E-07 | 0.999999 | 6.133E-07 |
| 15 | 3.681E-07 | 0.999999 | 6.818E-07 |
| 16 | 0.018129563 | 0 | 0.981870437 |
| 17 | 0.016312676 | 0 | 0.983687324 |
| 18 | 8.41E-08 | 0 | 0.999999916 |
| 19 | 2.828E-07 | 0.999999 | 5.519E-07 |
| 20 | 0.999995029 | 0 | 4.9711E-06 |
| 21 | 4.101E-07 | 0.999999 | 7.472E-07 |
| 22 | 5.0352E-06 | 0 | 0.999994965 |
| 23 | 1.9547E-06 | 0 | 0.999998045 |
| 24 | 0.999999495 | 0 | 5.046E-07 |
| 25 | 0.020529905 | 0 | 0.979470095 |
| 26 | 2.971E-07 | 0.999999 | 0.000000573 |
| 27 | 2.874E-07 | 0 | 0.999999713 |
| 28 | 4.746E-07 | 0.999999 | 8.474E-07 |
| 29 | 0.000942341 | 0 | 0.999057659 |
| 30 | 0.999975367 | 0 | 2.46329E-05 |
| 31 | 2.5E-09 | 0 | 0.999999998 |
| 32 | 7.02764E-05 | 0 | 0.999929724 |
| 33 | 7.9E-09 | 0 | 0.999999992 |
| 34 | 6.161E-07 | 0 | 0.999999384 |
| 35 | 0.999315619 | 0 | 0.000684381 |
| 36 | 2.668E-07 | 0.999999 | 5.293E-07 |
| 37 | 1E-10 | 0 | 1 |
| 38 | 0.999999993 | 0 | 7.5E-09 |
| 39 | 8.851E-07 | 0.999998 | 1.4725E-06 |
| 40 | 0.999455639 | 0 | 0.000544361 |
| 41 | 0 | 0 | 1 |
| 42 | 5.6E-09 | 0 | 0.999999994 |
| 43 | 0.000000008 | 0 | 0.999999992 |
| 44 | 0.999970973 | 0 | 2.90274E-05 |
| 45 | 3.6E-09 | 0 | 0.999999996 |
| 46 | 0 | 0 | 1 |
| 47 | 2.499E-07 | 0.999999 | 0.000000509 |
| 48 | 0.000000521 | 0.999999 | 9.192E-07 |
| 49 | 2.429E-07 | 0.999999 | 5.053E-07 |
| 50 | 0.000000017 | 0 | 0.999999983 |
| 51 | 1.3455E-06 | 0 | 0.999998655 |
| 52 | 0.999998083 | 0 | 1.9173E-06 |
| 53 | 3.9033E-06 | 0 | 0.999996097 |
| 54 | 4E-10 | 0 | 1 |
| 55 | 0.99999711 | 0 | 2.8903E-06 |
| 56 | 0.99940932 | 0 | 0.00059068 |
| 57 | 2E-10 | 0 | 1 |
| 58 | 5.364E-07 | 0.999999 | 9.429E-07 |
| 59 | 0.972458251 | 0 | 0.027541749 |
| 60 | 4.662E-07 | 0.999999 | 8.343E-07 |
| 61 | 1E-10 | 0 | 1 |
| 62 | 8.5E-09 | 0 | 0.999999992 |
| 63 | 1.2E-09 | 0 | 0.999999999 |
| 64 | 0 | 0 | 1 |
| 65 | 0.000000004 | 0 | 0.999999996 |
| 66 | 4.169E-07 | 0.999999 | 7.577E-07 |
| 67 | 3.198E-07 | 0.999999 | 6.073E-07 |
| 68 | 1E-10 | 0 | 1 |
| 69 | 1.9757E-06 | 0 | 0.999998024 |
| 70 | 3.05E-08 | 0 | 0.99999997 |
| 71 | 3E-10 | 0 | 1 |
| 72 | 3E-10 | 0 | 1 |
| 73 | 1E-10 | 0 | 1 |
| 74 | 0 | 0 | 1 |
| 75 | 1.8E-09 | 0 | 0.999999998 |
| 76 | 9E-10 | 0 | 0.999999999 |
| 77 | 2.98E-08 | 0 | 0.99999997 |
| 78 | 3.43E-08 | 0 | 0.999999966 |
| 79 | 0.000000006 | 0 | 0.999999994 |
| 80 | 1E-10 | 0 | 1 |
| 81 | 0.000001411 | 0.999996 | 2.2467E-06 |
| 82 | 2.0191E-06 | 0.999995 | 3.1177E-06 |
| 83 | 0.999999854 | 0 | 1.459E-07 |
| 84 | 3.309E-07 | 0.999999 | 6.244E-07 |
| 85 | 0.999999973 | 0 | 2.68E-08 |
| 86 | 0.998605059 | 0 | 0.001394942 |
| 87 | 0.99999974 | 0 | 2.601E-07 |
| 88 | 0.999999999 | 0 | 1.1E-09 |
| 89 | 0.999263245 | 0 | 0.000736755 |
| 90 | 0.999582242 | 0 | 0.000417758 |
| 91 | 2.2544E-06 | 0.999994 | 3.4495E-06 |
| 92 | 1 | 0 | 0 |
| 93 | 2.522E-07 | 0.999999 | 5.552E-07 |
| 94 | 0.999180319 | 0 | 0.000819681 |
| 95 | 0.999658964 | 0 | 0.000341036 |
| 96 | 1.0646E-06 | 0.999997 | 1.7397E-06 |
| 97 | 1.3281E-06 | 0.999997 | 2.1262E-06 |
| 98 | 0.999998501 | 0 | 1.4988E-06 |
| 99 | 0.999908192 | 0 | 9.18085E-05 |
| 100 | 0.999987621 | 0 | 1.23795E-05 |
| 101 | 3.231E-07 | 0.999999 | 7.572E-07 |
| 102 | 0.000000291 | 0.999999 | 0.000000564 |
| 103 | 2.604E-07 | 0.999999 | 5.209E-07 |
| 104 | 0.999999152 | 0 | 8.476E-07 |
| 105 | 4.641E-07 | 0.999999 | 0.000000831 |
| 106 | 0.999999532 | 0 | 4.681E-07 |
| 107 | 9.755E-07 | 0.999997 | 1.6075E-06 |
| 108 | 0.999998837 | 0 | 1.1635E-06 |
| 109 | 0.999577117 | 0 | 0.000422884 |
| 110 | 0.999999202 | 0 | 0.000000798 |
| 111 | 3.169E-07 | 0.999999 | 6.029E-07 |
| 112 | 0.997298409 | 0 | 0.002701592 |
| 113 | 8.148E-07 | 0.999998 | 0.000001367 |
| 114 | 2.9165E-06 | 0.999993 | 4.3717E-06 |
| 115 | 4.274E-07 | 0.999999 | 7.741E-07 |

**Supplementary table 2:** Post-hoc pairwise PERMANOVA testing for body structures (clypeus, protibia, elytra, and wing) between females and trimorphic males (alpha, beta, and gamma) of *Oxysternon palemo* (Scarabaeinae: Phanaeini).

| **Structures** | **Groups** | **Female** | **Alpha** | **Beta** | **Gamma** |
| --- | --- | --- | --- | --- | --- |
| Clypeus | Female | - | 0.0001* | 0.0001* | 0.0001* |
|  | Alpha | 0.0001* | - | 0.0001* | 0.0001* |
|  | Beta | 0.0001* | 0.0001* | - | 0.0004* |
|  | Gamma | 0.0001* | 0.0001* | 0.0004* | - |
|  |  |  |  |  |  |
| Protibia | Female | - | 0.0001* | 0.0001* | 0.0001* |
|  | Alpha | 0.0001* | - | 0.0001* | 0.0001* |
|  | Beta | 0.0001* | 0.0001* | - | 0.0001* |
|  | Gamma | 0.0001* | 0.0001* | 0.0001* | - |
|  |  |  |  |  |  |
| Elytra | Female | - | 0.0001* | 0.0001* | 0.0001* |
|  | Alpha | 0.0001* | - | 0.0001* | 0.0001* |
|  | Beta | 0.0001* | 0.0001* | - | 0.0001* |
|  | Gamma | 0.0001* | 0.0001* | 0.0001* | - |
|  |  |  |  |  |  |
| Wing | Female | - | 0.0001* | 0.0001* | 0.0001* |
|  | Alpha | 0.0001* | - | 0.0063* | 0.0001* |
|  | Beta | 0.0001* | 0.0063* | - | 0.1853 |
|  | Gamma | 0.0001* | 0.0001* | 0.1853 | - |

**Supplementary table 3:** Post-hoc Tukey test between females and trimorphic males (alpha, beta, gamma) for mixed-effects linear models (GLMM) of biomechanical indices: Wing Aspect Ratio (WAR), Wing Loading (WL), and Wing Loading Moment (WLM).

| **Structures** | **contrast** | **estimate** | **SE** | **df** | **t.ratio** | **p.value** |
| --- | --- | --- | --- | --- | --- | --- |
| WAR | alpha - beta | -0.1267 | 0.155 | 149 | -0.818 | 0.8459 |
|  | alpha - female | -0.0605 | 0.144 | 149 | -0.419 | 0.9752 |
|  | alpha - gamma | 0.5260 | 0.158 | 149 | 3.327 | 0.0060* |
|  | beta - female | 0.0662 | 0.162 | 149 | 0.408 | 0.9770 |
|  | beta - gama | 0.6527 | 0.163 | 149 | 3.995 | 0.0006* |
|  | female - gama | 0.5864 | 0.165 | 149 | 3.550 | 0.0029* |
|  |  |  |  |  |  |  |
| WL | alpha - beta | 2.22e-04 | 0.000129 | 149 | 1.715 | 0.3195 |
|  | alpha - female | 8.71e-05 | 0.000123 | 149 | 0.706 | 0.8945 |
|  | alpha - gamma | 5.31e-04 | 0.000132 | 149 | 4.034 | 0.0005* |
|  | beta - female | -1.34e-04 | 0.000135 | 149 | -0.996 | 0.7520 |
|  | beta - gama | 3.09e-04 | 0.000143 | 149 | 2.168 | 0.1370 |
|  | female - gama | 4.44e-04 | 0.000137 | 149 | 3.231 | 0.0082* |
|  |  |  |  |  |  |  |
| WLM | alpha - beta | 0.02344 | 0.00704 | 149 | 3.329 | 0.0060* |
|  | alpha - female | 0.00664 | 0.00672 | 149 | 0.987 | 0.7570 |
|  | alpha - gamma | 0.04372 | 0.00717 | 149 | 6.097 | <0.0001* |
|  | beta - female | 0.01680 | 0.00736 | 149 | -2.283 | 0.1066 |
|  | beta - gama | 0.02028 | 0.00777 | 149 | 2.610 | 0.0486* |
|  | female - gama | 0.03708 | 0.00748 | 149 | 4.956 | <0.0001* |
|  |  |  |  |  |  |  |
